# MicroRNA-92a Inhibition Attenuates Hypoxia/Reoxygenation-Induced Myocardiocyte Apoptosis by Targeting *Smad7*

**DOI:** 10.1371/journal.pone.0100298

**Published:** 2014-06-18

**Authors:** Busheng Zhang, Mi Zhou, Canbo Li, Jingxin Zhou, Haiqing Li, Dan Zhu, Zhe Wang, Anqing Chen, Qiang Zhao

**Affiliations:** Department of Cardiac Surgery, Ruijin Hospital, Shanghai Jiaotong University School of Medicine, Shanghai, China; Georgia Regents University, United States of America

## Abstract

**Background:**

MicroRNAs (miRNAs) regulate a lot of physiological and pathological processes, including myocardial ischemia/reperfusion. Recent studies reported that knockdown of miR-92a could attenuate ischemia/reperfusion-induced myocardial injury. In the present study, we examined the potential anti-apoptotic effects of miR-92a in a rat myocardiocyte cell line, and the possible role of *Smad7* in such actions.

**Methodology and Results:**

In a preliminary bioinformatic analysis, we identified *SMAD family member 7* (*Smad7*) as a potential target for miR-92a. A luciferase reporter assay indeed demonstrated that miR-92a could inhibit *Smad7* expression. Myocardial ischemia/reperfusion was simulated in rat H9c2 cells with 24-h hypoxia followed by 12-h reoxygenation. Prior to hypoxia/reoxygenation, cells were transfected by miR-92a inhibitor. In some experiments, cells were co-transfected with siRNA-Smad7. The miR-92a inhibitor dramatically reduced the release of lactate dehydrogenase and malonaldehyde, and attenuated cardiomyocyte apoptosis. The miR-92a inhibitor increased SMAD7 protein level and decreased nuclear NF-κB p65 protein. Effects of the miR-92a inhibitor were attenuated by co-transfection with siRNA-Smad7.

**Conclusion:**

Inhibiting miR-92a can attenuate myocardiocyte apoptosis induced by hypoxia/reoxygenation by targeting *Smad7*.

## Introduction

Myocardial ischemia/reperfusion (I/R) injury contributes to the damage after ischemic events in patients with coronary heart disease (CHD) [1], [2]. I/R injury is also implicated in cardiac procedures that require cardio-pulmonary bypass, and in CHD patients receiving percutaneous coronary intervention or coronary artery bypass surgery. I/R injury is mediated by a variety of factors, including oxidative stress, intracellular Ca^2+^ overload, rapid restoration of physiological pH upon reperfusion, the mitochondrial permeability transition pore (MPTP), and exaggerated inflammation [3].

MicroRNAs (miRNAs) are a class of endogenous, small non-coding single-stranded RNAs, typically 18–24 nucleotides in length, that negatively regulate gene expression through binding to the 3′-untranslated region (UTR) of target mRNAs [4]. MiRNAs play critical roles in a variety of heart diseases, including cardiac hypertrophy [5], heart failure [6], arrhythmia [7], myocardial infarction [8] and I/R injury [9]. Growing evidence also supports a pivotal role for miR-92a in multiple processes, including tumorigenesis and metastasis [10], cell proliferation and apoptosis [11]. In the study, we found that transfection with miR-92a inhibitor could attenuate myocardial injury and apoptosis induced by hypoxia/reoxygenation (H/R) in cultured rat H9c2 myocardiocytes cells. A preliminary bioinformatics analysis identified *Smad7* as a target for miR-92a. Accordingly, we also examined the possible involvement of *Smad7* in the protective action of miR-92a.

## Materials and Methods

### Cell Culture

The H9c2 cells (ventricular myocardiocyte, rat in origin; Cell Bank of the Chinese Academy of Sciences, Shanghai, China) were seeded at a density of 2×10^4^ cells/cm^2^ in 6-well plates and cultured in Dulbecco’s modified Eagle’s medium (DMEM, Sigma, St. Louis, MO, USA) containing 10% (v/v) fetal bovine serum (FBS, HyClone, Logan, UT, USA) in a humidified atmosphere of 95% air and 5% CO_2_ at 37°C.

### Transient Transfection with Oligonucleotides

Transfection was carried out using Lipofectamine 2000 (Invitrogen, Carlsbad, CA, USA). The ratio of oligonucleotide vs. the Lipofectamine 2000 transfection reagent was 1∶5. MiR-92a mimic, inhibitor and matched negative control (NC) were synthesized by GenePharma, Shanghai, China. For RNA interference, cells were transiently transfected with a siRNA specific for *Smad7* or NC (GenePharma). All transfections were carried out after 12-h serum starvation, and lasted for 48-h prior to the H/R experiments.

### H/R in H9c2 Cardiomyocytes

Hypoxia was induced by exposing the cells to 1% O_2_, 94% N_2_, and 5% CO_2_ for 24 h using a modular incubator (Model 3131, Forma Scientific, Marietta, OH, USA). Reoxygenation (95% air, 5% CO_2_, 37°C) lasted for 12 h. Cells under normoxia throughout the experiments were included as a control. All experiments were repeated three times.

### Quantitative Real-time Polymerase Chain reaction (qRT-PCR)

Total RNA was extracted using Trizol reagent (Invitrogen). Bulge-loop miRNA qRT-PCR primer sets (one RT primer and a pair of qRT-PCR primers for each set) specific for miR-17, miR-18a, miR-19a, miR-20a, miR-19b and miR-92a were designed by RiboBio (Guangzhou, China). MiRNAs were reverse transcribed using the stem-loop RT primer. The primers for Smad7 were also designed by RiboBio. qRT-PCR was carried out to examine the expression of specific miRNAs or mRNA on a Rotor-Gene 3,000 real-time DNA detection system (Corbett Research, Sydney, Australia) using SYBR Green (Qiagen, Shanghai, China). All samples were analyzed in triplicate. Gene expression was determined by comparing the data against the standard curve, and normalized against U6.

### Determination of Cell Injury and Apoptosis

Structural integrity of cultured H9c2 cardiomyocytes was evaluated by measuring the concentration of lactate dehydrogenase (LDH) and malonaldehyde (MDA) in the culture media by ELISA using an automatic biochemical analyzer (Model 7150, Hitachi, Tokyo, Japan). Apoptosis was detected by annexinV-FITC/propidium iodide (AV/PI) dual staining (Bender MedSystems, Burlingame, CA, USA).

### DNA Constructs and Reporter Gene Assays

To examine whether miR-92a regulates the expression of *Smad7*, we used a dual luciferase psiCheck-2 reporter plasmid (Promega, Madison, WI, USA) to generate a reporter plasmid harboring the *Smad7* 3′-UTR. For luciferase reporter experiments, the 3′UTR of the *Smad7* gene was amplified by PCR from rat genomic DNA and cloned into psiCHECK-2 (Promega) between the *Not*1 and *Sgf*1 sites. The construct with a mutated targeting fragment (TATACCG) in the 3′-UTR of *Smad7* lacking the putative miR-92a binding sequence was used as a mutated control. 293T cells were co-transfected with psiCheck2 containing the *Smad7* 3′-UTR and the miR-92a mimic using Lipofectamine 2000 (Invitrogen). Co-transfection with non-targeting negative control RNA was performed as a control. The cells were harvested 24 h after transfection for luciferase activity using a dual luciferase reporter assay kit (Promega) on a luminometer (Lumat LB9507).

### Immunocytochemistry

Cultured H9c2 cardiomyocytes were fixed in 4% paraformaldehyde and permeabilized with 0.1% Triton. Cells were blocked with 3% BSA and incubated with 1000-fold diluted primary antibody against SMAD7 (ab90085; Abcam; Cambridge, MA, USA) overnight, and then stained with fluorochrome- conjugated secondary antibody for another 60 min. Cells were mounted in Vectashield mounting medium containing 4′,6′-diamidino-2-phenylindole (DAPI) to visualize nuclei. Images were captured using a fluorescence laser scanning confocal microscope (FV1000, Olympus, Tokyo, Japan).

### Western Blotting Assays

Cells were harvested in RIPA lysis buffer (Bioteke Co, Beijing, China) containing 1 mM phenylmethylsulfonyl fluoride and centrifuged at 12,000×g for 15 min at 4°C. Whole cell lysate was used for SMAD7 detection. Cytosolic and nuclear fractions were prepared using standard nuclear and cytoplasmic extraction reagents (Thermo Scientific, Rockford, IL, USA). Protein concentration was measured using the Bio-Rad method. Samples (20 µg protein) were separated by 10% SDS-PAGE and transferred to a nitrocellulose membrane. The membranes were blocked with 5% non-fat milk in TBST buffer (100 mM NaCl, 10 mM Tris-HCl, pH 7.4, 0.1% Tween-20) for 1 h prior to incubation with a primary antibody against SMAD7 (1∶1000; ab90085; Abcam), NF-κB p65 (1∶1000; ab7970; Abcam), GAPDH (1∶2500; ab7970; Abcam) or lamin B1 (1∶1000; #13435; Cell Signaling Technology; Boston, MA, USA) at 4°C overnight, followed by incubation with an appropriate peroxidase-conjugated secondary antibody (1∶1000 dilution). Signal was visualized by chemiluminescence (Odyssey Li-COR) using GAPDH as a control. In the case of nuclear NF-κB p65, lamin B1 was employed as the loading control. Band intensity was assessed using Quantity one 4.6.2 software.

### Statistics and Data Analysis

All data are expressed as the mean±SEM. Comparisons between groups were made by one-way analysis of variance or two-tailed student’s *t*-test. Differences were considered statistically significant at *P*<0.05. SPSS software version 19.0 (SPSS, Chicago, IL, USA) was used for data analysis. All experiments were performed at least three times.

## Results

### MiR-17-92 Expression Profiles in H/R H9c2 Cardiomyocytes

**Figure 1:**
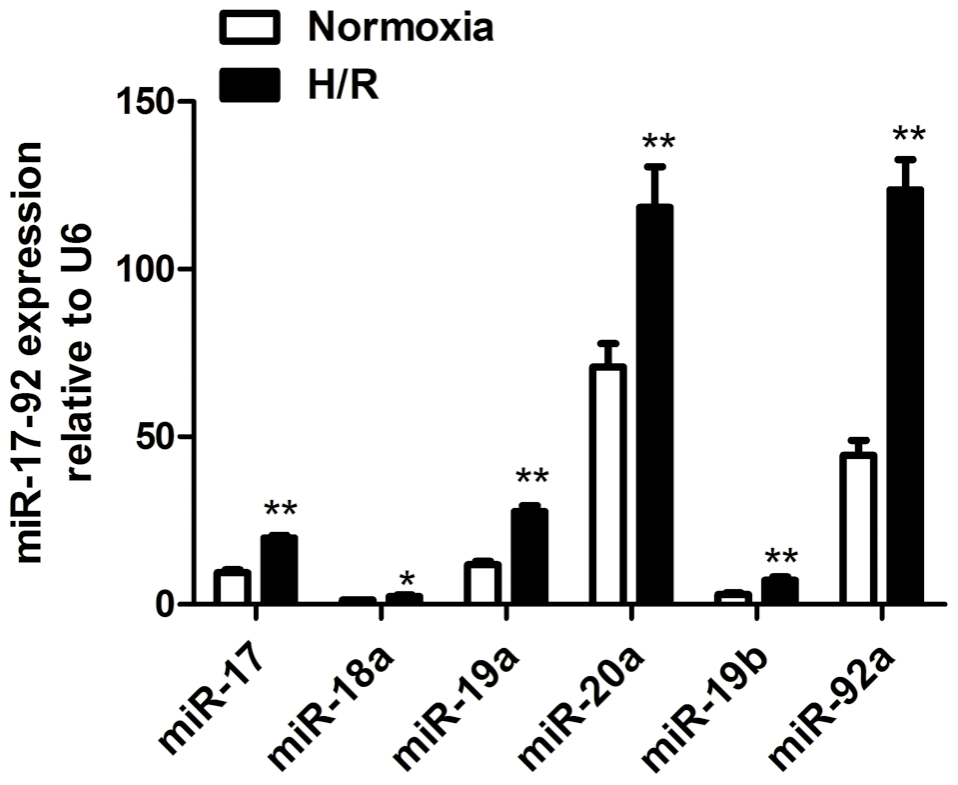
The expression level of the miR-17-92 cluster in H9c2 cells by qRT-PCR. Fold changes of miR-17, miR-18a, miR-19a, miR-20a, miR-19b, and miR-92a are shown in the H/R group compared with normoxic controls (normalized to U6), respectively, *P<0.05, **P<0.01. H/R, hypoxia/reoxygenation.

In our previous study [12], we found that miR-17, miR-19a, miR-20a, miR-19b and miR-92a, but not miR-18a, were highly expressed in the heart of C57BL/6 mice. In the current study, the expression of the miR-17-92 cluster was up-regulated in H/R H9c2 cardiomyocytes: the expression of miR-92a was significantly up-regulated by 2.78-fold over the control (*P*<0.01 *vs.* control) (Figure 1). Based on the most remarkable change in response to hypoxia/reoxygenation as reflected by qRT-PCR, miR-92a was selected for subsequent experiments.

### Efficiency of RNA Interference

**Figure 2:**
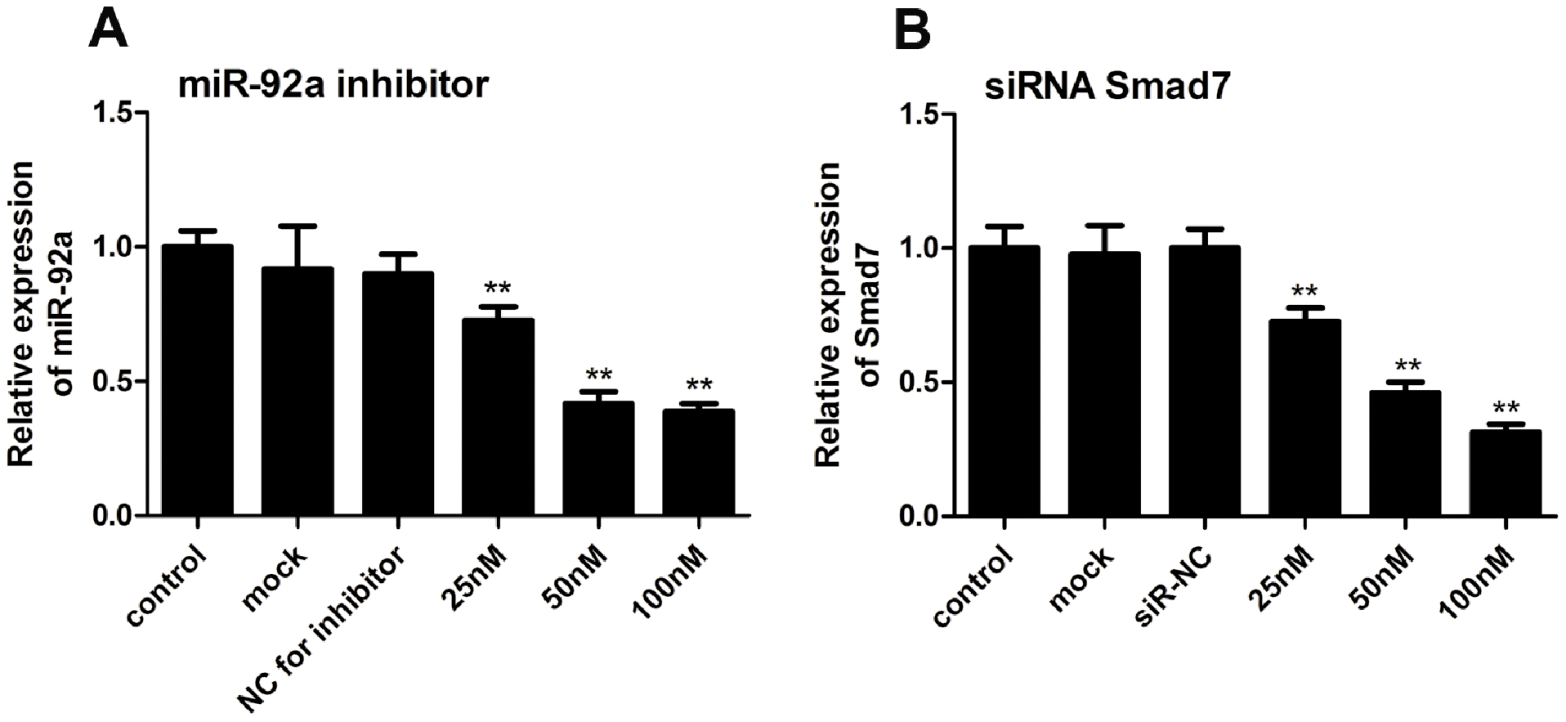
Gene levels in normoxic H9c2 cardiomyocytes transfected with miR-92a inhibitor (A) or siRNA-Smad7 (B). H9c2 cells were transfected with miR-92a inhibitor or siRNA-Smad7 with Lipofectamine2000 for 2 days. The cells were then harvested for measurement. Mock transfection (transfection agent without RNA) and non-targeting negative control were used as controls. The expression levels of miR-92a and *Smad7* mRNA were determined using qRT-PCR, normalized to U6, and expressed as the fold change relative to the control (**P<0.01 *vs.* control).

Transfection of miR-92a inhibitor significantly decreased the level of miR-92a in cultured H9c2 cells under normoxic conditions, respectively (Figure 2A). At 50 nM, the miR-92a inhibitor significantly down-regulated miR-92a by 2.43±0.06-fold (*P*<0.01 *vs.* control). Neither mock nor NC RNA transfection affected miR-92a expression under normoxic cultures. Based on such preliminary experiments, 50 nM was chosen for subsequent experiments. At 100 nM, siRNA-Smad7 significantly decreased *Smad7* expression by 3.32±0.13-fold (*P*<0.01 *vs.* control) (Figure 2B).

### Inhibition of miR-92a Protects against H/R-induced Injury and Apoptosis

**Figure 3:**
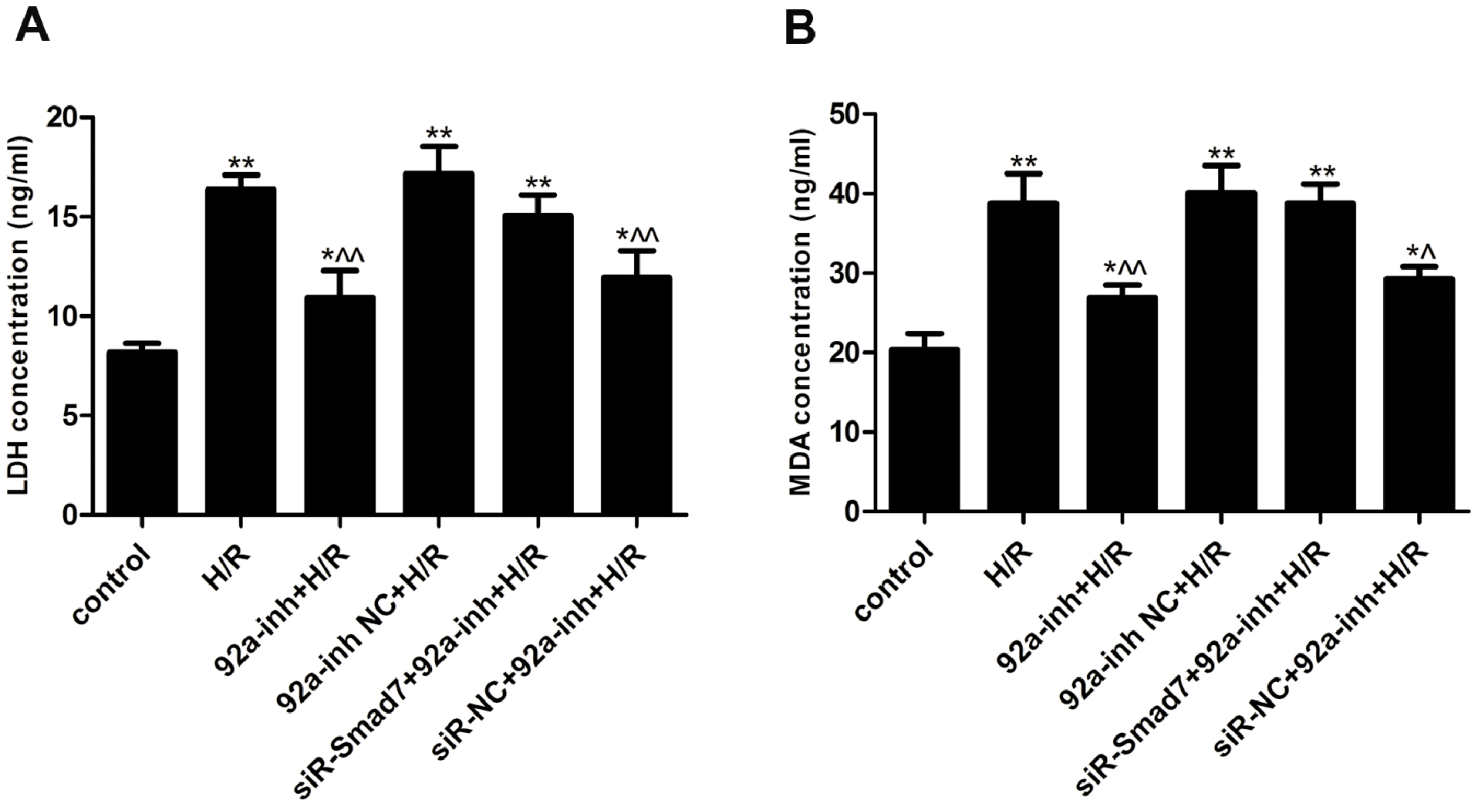
Cell injuries were determined in H9c2 cardiomyocytes. A. Lactate dehydrogenase (LDH) release. B. Malonaldehyde (MDA) release. Data are presented as mean±SEM from three independent experiments (*P<0.05 and **P<0.01 *vs.* the control group; ∧*P*<0.05 and ∧∧*P*<0.01 *vs.* the H/R group). H/R, hypoxia/reoxygenation.

H/R treatment increased LDH in the culture media (16.36±0.74 *vs.* 8.16±0.47 ng/mL in normoxic condition, *P*<0.01) (Figure 3A). The miR-92a inhibitor significantly decreased LDH release in response to H/R (10.93±1.35 ng/mL, *P*<0.01 *vs.* the H/R group). Co-transfection with siRNA-Smad7 attenuated the effects of the miR-92a inhibitor.

H/R treatment increased MDA release (38.83±3.70 *vs.* 20.33±2.05 ng/mL in normoxic condition, *P*<0.01) (Figure 3B). The H/R-induced MDA release was significantly decreased by the miR-92a inhibitor (26.93±1.59 ng/mL, *P*<0.01 *vs.* the H/R group). The observed effects of the miR-92a inhibitor were also attenuated by co-transfection with siRNA-Smad7.

**Figure 4:**
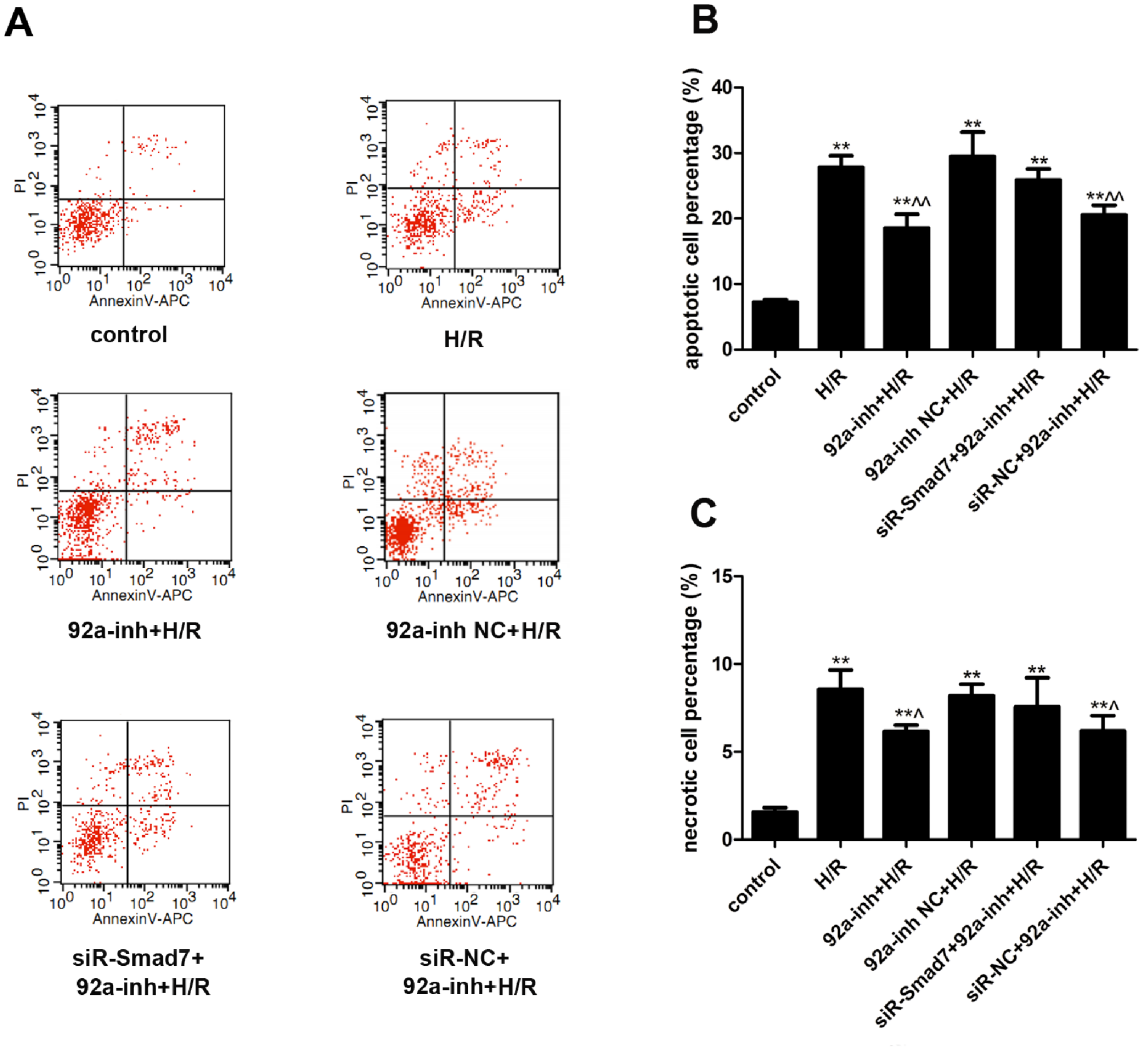
Cell death was determined in H9c2 cardiomyocytes. A. Representative dot-plot diagrams of AV/PI flow cytometry; B. Apoptotic cell percentage; C. Necrotic cell percentage. Data are presented as mean±SEM from three independent experiments (*P<0.05 and **P<0.01 *vs.* the control group; ∧*P*<0.05 and ∧∧*P*<0.01 *vs.* the H/R group). H/R, hypoxia/reoxygenation.

The AV/PI dual staining (Figure 4A) revealed increased apoptosis upon H/R (27.80±1.77% *vs.* 7.23±0.40% under normoxic condition, *P*<0.01) (Figure 4B). Transfection with miR-92a inhibitor significantly decreased the percentage of apoptosis induced by H/R (18.56±2.08%, *P*<0.01 *vs.* the H/R group). The effects of miR-92a inhibitor were attenuated by co-transfection with siRNA-Smad7.

H/R treatment also significantly increased the percentage of necrotic cells (8.56±1.10 *vs.* 1.56±0.25% in the control; *P*<0.01) (Figure 4C). Transfection with miR-92a inhibitor significantly decreased the percentage of necrosis induced by H/R (6.16±0.35%, *P*<0.05 *vs.* the H/R group). The effects of miR-92a inhibitor were also attenuated by co-transfection with siRNA-Smad7.

### *Smad7* is a Target of miR-92a

**Figure 5:**
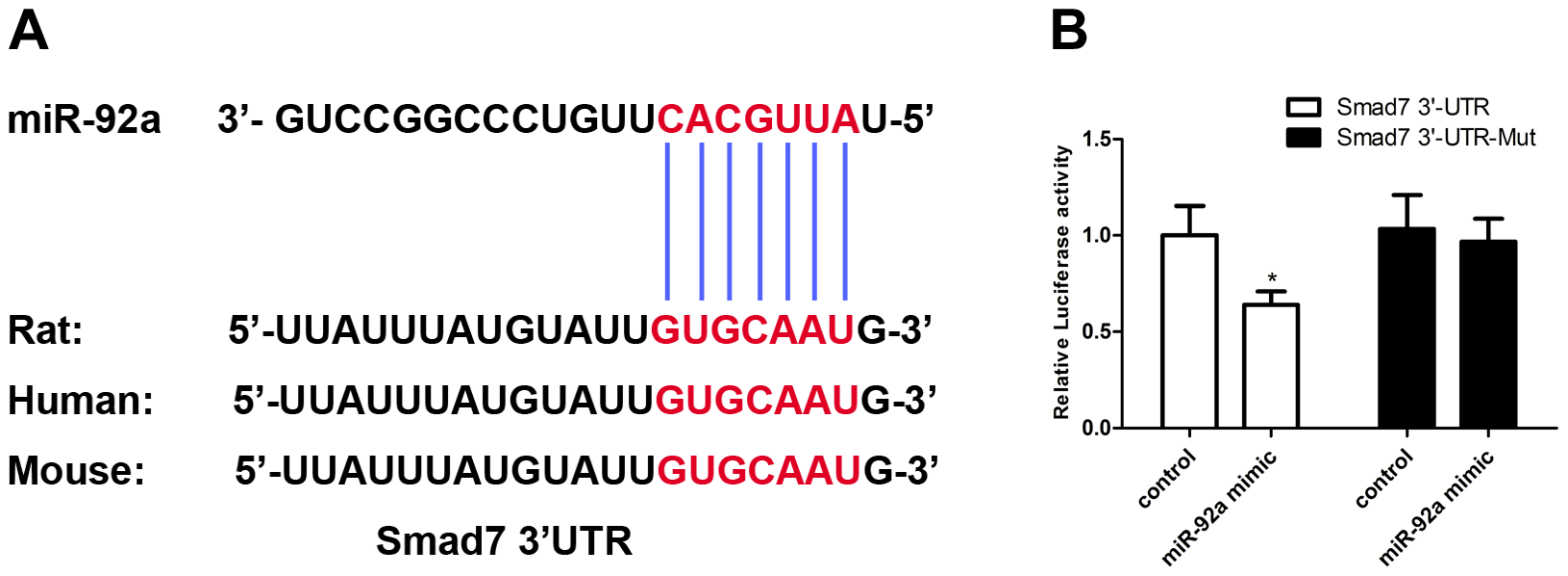
MiR-92a directly regulates *Smad7* expression via 3′-UTR site. A. The potential binding site for miR-92a in the 3′-UTR of *Smad7* mRNA. The complementary nucleotides between miR-92a and the target region of *Smad7* 3′-UTR are indicated with short vertical lines. B. Luciferase reporter assay was performed by co-transfection of 293T cells with luciferase reporter containing the 3′-UTR of rat *Smad7* with miR-92a mimic. Luciferase activity was determined 24 h after transfection. Data are presented as mean±SEM from three independent experiments (*P<0.05 *vs.* the control group).

Bioinformatic analysis using MiRanda, miRDB, miRwalk and TargetScan suggested *Smad7* as a target of miR-92a. Specifically, the 3′-UTR of the *Smad7* mRNA contains one binding site for miR-92a (Figure 5A).

**Figure 6:**
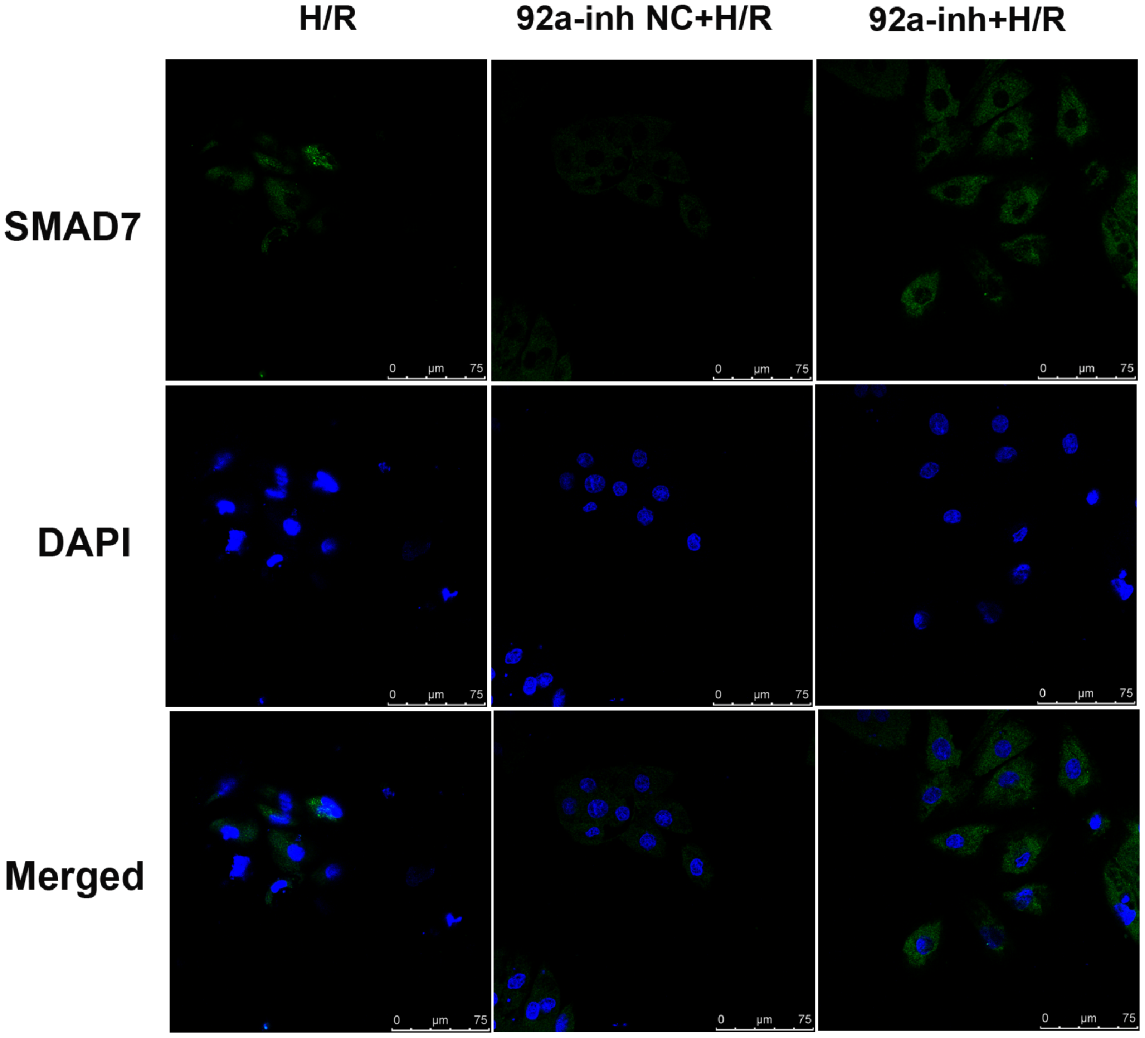
The effect of miR-92a on SMAD7 was observed by immunocytofluorescent staining. H9c2 cells were plated in 24-well plates and cultured to 80–90% confluence for transient transfection with the miR-92a inhibitor (50 nM) or NC (50 nM), respectively. Immunocytofluorescence analysis was performed 72 h after transfection. Bar: 75 µm. H/R, hypoxia/reoxygenation.

In comparison with the mutated control, the miR-92a mimic reduced the activity of the luciferase reporter fused with the *Smad7* 3′-UTR by 41% (Figure 5B). Inmunocytofluorescent staining (Figure 6) revealed very low level of SMAD7 in cells exposed to the H/R treatment. The protein level of SMAD7 was increased by the miR-92a inhibitor.

**Figure 7:**
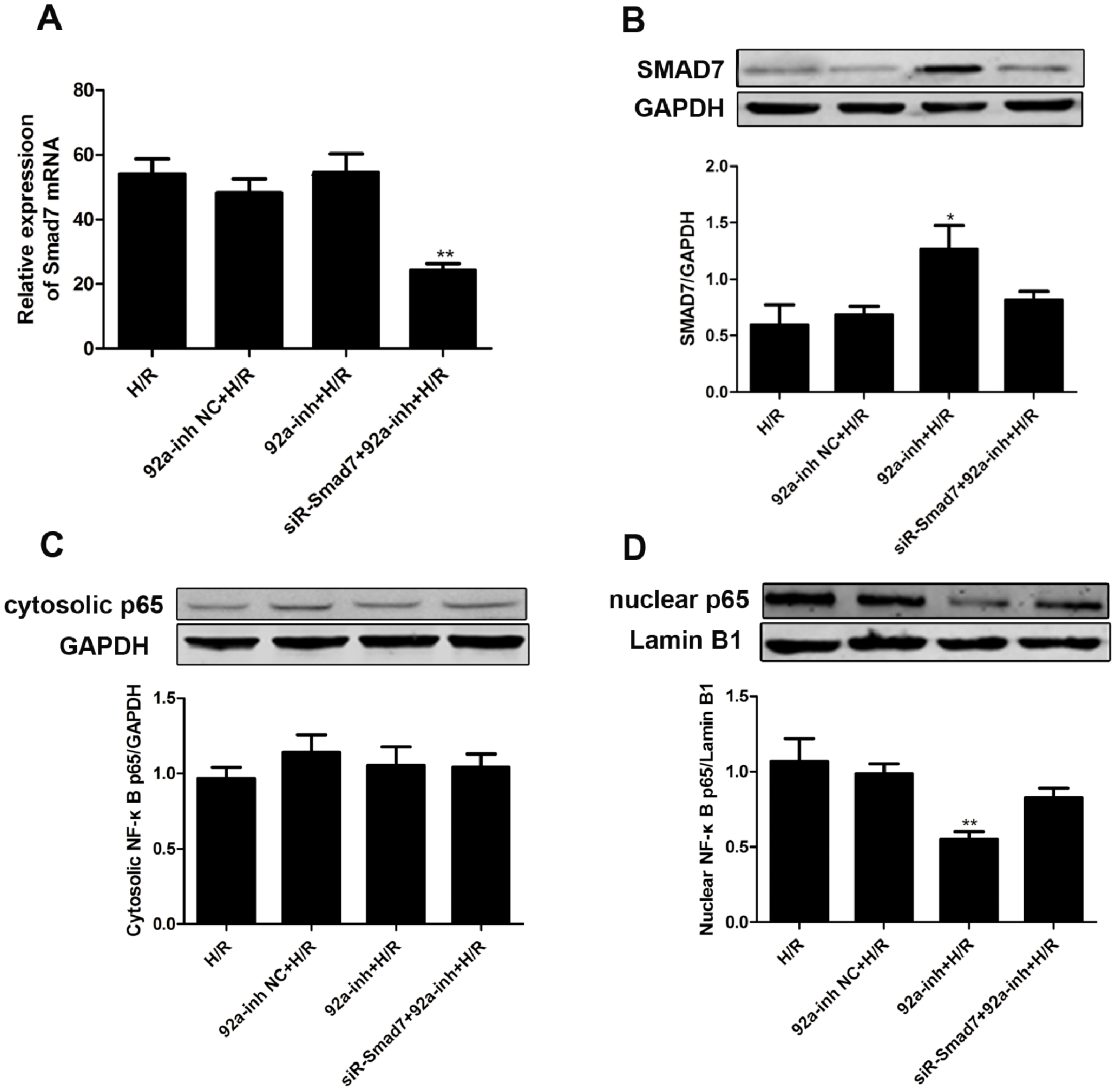
Inhibition of miR-92a promotes SMAD7 expression and activates the Smad7/NF-κB signaling pathway. A. qRT-PCR analysis of endogenous *Smad7* mRNA levels in the cardiomyocytes transfected with miR-92a inhibitor, or co-transfected with miR-92a inhibitor and siRNA-Smad7. B. Western blotting assays for the SMAD7 protein level in the cardiomyocytes transfected with miR-92a inhibitor, or co-transfected with miR-92a inhibitor and siRNA-Smad7. C. Western blotting assays for the cytosolic NF-κB p65 protein levels in the cardiomyocytes transfected with miR-92a inhibitor, or co-transfected with miR-92a inhibitor and siRNA-Smad7. D. Western blotting assays for the nuclear NF-κB p65 protein levels in the cardiomyocytes transfected with miR-92a inhibitor, or co-transfected with miR-92a inhibitor and siRNA-Smad7. (*P<0.05 and **P<0.01 *vs.* the H/R group). H/R, hypoxia/reoxygenation.

The miR-92a inhibitor did not affect the level of *Smad7* mRNA (Figure 7A). Co-transfection with siRNA-Smad7 significantly decreased the level of *Smad7* mRNA (*P*<0.01). Western blotting (Figure 7B) revealed increased level of SMAD7 by the miR-92a inhibitor (in comparison to H/R alone, *P*<0.05). The effects of miR-92a inhibitor were attenuated by co-transfection with siRNA-Smad7.

Cytosolic NF-κB p65 was not affected by transfection with miR-92a inhibitor, or co-transfection with miR-92a inhibitor and siRNA-Smad7 (Figure 7C). Nuclear NF-κB p65 was significantly decreased by the miR-92a inhibitor (in comparison with H/R alone, *P*<0.05). The effects of the miR-92a inhibitor on nuclear NF-κB p65 were attenuated by co-transfection with siRNA-Smad7 (Figure 7D).

## Discussion

Apoptosis plays a crucial role in myocardial I/R injury [13], [14]. A number of miRNAs, including miR-1 [15], miR-15b [16], miR-21 [17] and miR-145 [18], have been implicated in myocardial I/R injury due to their effects on key genes associated with apoptosis. MiR-92a has been implicated in myocardial I/R injury in variety of experimental models. Bonauer et al. demonstrated that the expression level of miR-92a was up-regulated 24 h after coronary artery ligation in mice [19]. They also showed that injection of antagomir-92a after permanent coronary artery ligation in mice improved left ventricular function, reduced myocardial infarction size and apoptosis, and increased the number of new blood vessels, especially in the border areas of the infarction. Hinkel and colleagues demonstrated that inhibiting miR-92a protects against myocardial I/R injury in a porcine model [20].

Studies of miRNAs in various models of myocardial I/R injury [21]–[24] indicated varying changes of miRNA expression across different species, indicating the complexity of miRNA responses, as well as the complexity of miRNA functions. For example, Hinkel et al demonstrated that inhibition of miR-92a significantly reduced I/R-induced cell apoptosis and necrosis in HL-1 cells [20]. Conversely, Bonauer et al. reported antagomir-92a did not affect cell apoptosis induced by I/R in cultured neonatal ventricular cardiomyocytes from Wistar rats [19]. Zhang et al. demonstrated that both overexpression and down-regulation of miR-92a could have pro-angiogenic effects in human umbilical endothelial cells (HUVEC) [25].

In the current study, cultured H9c2 cardiomyocytes were subjected to 24-h hypoxia followed by 12-h reoxygenation. qRT-PCR analysis revealed increased expression of all miRNAs in the miR-17-92 cluster upon H/R treatment. Increased expression of miR-92a was the most prominent at 2.78-fold.

The present study showed that the inhibition of miR-92a could significantly reduce H/R-induced myocardiocyte injury and apoptosis. Based on bioinformatic analyses, *Smad7* was identified as a target of miR-92a. Such a prediction was confirmed by a dual luciferase reporter assay.

Through imperfect sequence-specific binding to the 3′-UTR of target mRNAs, miRNAs down-regulate gene expression by degrading target mRNAs [26], [27] and/or inhibiting translation [28]. The present study demonstrated that inhibition of miR-92a significantly increased protein levels of SMAD7, but did not affect *Smad7* mRNA levels, indicating that miR-92a inhibits the protein translation at the post-transcriptional level, but does not promote Smad7 mRNA degradation.

SMAD7 is an important transcriptional factor that regulates the expression of apoptosis-related genes involved in myocardial I/R injury [29], [30]. SMAD7 protects against apoptosis through inhibiting the NF-κB signaling pathway [31], [32]. Put together, our findings suggest that apoptosis in myocardial I/R injury, is mediated, at least partly, through the miR-92a/Smad7/NF-κB p65 pathway.

Despite of our findings, whether *Smad7* is the most important target of miR-92a in cardiomyocytes (and thus the therapeutic potentials) remains unknown. Other potential candidates included (but not limited to) Pten and MKK4 [33], [34] Also, the current study did not provide direct evidence for the interaction between *Smad7* and NF-κB p65. Another limitation of the current study is the use of myocardiocytes from a single species (rats), without the presence of endothelial and inflammatory cells. Based on the Hinkel et al. study that demonstrated reduced I/R-induced cell apoptosis and necrosis in HL-1 cells upon miR-92a inhibition in a murine myocyte-like cell line [20], we boldly speculate that our findings may be generalized to other species although such a generalization clearly needs to be verified.

Taken together, the current study indicated that inhibition of miR-92a can attenuate cardiomyocyte apoptosis induced by H/R via the up-regulation of SMAD7 and down-regulation of nuclear NF-κB p65.
